# Biotransformation of Food-Grade and Nanometric TiO_2_ in the Oral–Gastro–Intestinal Tract: Driving Forces and Effect on the Toxicity toward Intestinal Epithelial Cells

**DOI:** 10.3390/nano10112132

**Published:** 2020-10-27

**Authors:** Arianna Marucco, Marion Prono, David Beal, Enrica Alasonati, Paola Fisicaro, Enrico Bergamaschi, Marie Carriere, Ivana Fenoglio

**Affiliations:** 1 Department of Chemistry, University of Torino, 10125 Torino, Italy; ariannamaria.marucco@unito.it; 2 Chimie Interface Biologie pour l’Environnement, la Santé et la Toxicologie (CIBEST), University Grenoble Alpes, CEA, CNRS, IRIG, SyMMES, F-38000 Grenoble, France; marion.prono@cea.fr (M.P.); david.beal@cea.fr (D.B.); 3 Département Biomédicale et Chimie Inorganique, Laboratoire National de Métrologie et D’essais, F-75724 Paris, France; Enrica.Alasonati@lne.fr (E.A.); Paola.Fisicaro@lne.fr (P.F.); 4 Department of Public Health and Pediatrics, University of Torino, 10126 Torino, Italy; enrico.bergamaschi@unito.it

**Keywords:** food, TiO_2_, intestinal cells, size, surface, bio-corona, toxicity

## Abstract

*Background:* Oral exposure to titanium dioxide (TiO_2_) is common since it is widely used in food and pharmaceutical products. Concern on the safety of this substance has been recently raised, due to the presence of an ultrafine fraction in food-grade TiO_2_. Discrepancy exists among data reported in in vitro and in vivo studies on intestinal acute/chronic toxicity of TiO_2_. This might be due to the different biological identity of TiO_2_ in traditional in vitro test by respect in vivo conditions. *Methods:* One food-grade TiO_2_ and two nanometric TiO_2_ samples were treated with a simulated human digestive dystem (SHDS) in order to investigate the bio-transformation occurring to the particles once ingested in term of size distribution (Dynamic Light Scattering—DLS-, Flow Particle Imaging, Asymmetric Flow Field Flow Fractionation-AF4-) and surface modification (Electrophoretic Light Scattering—ELS-, Electron Paramagnetic Resonance Spectroscopy—EPR-). The effect of SHDS on the cyto-, genotoxicity and potential to induce oxidative stress towards human colorectal carcinoma HCT116 cells was also assessed. *Results:* Aggregation as a consequence of the high ionic strength of the gastric and intestinal simulated fluids was observed, together with the formation of a partially irreversible bio-corona containing phosphate ions and proteins. Such bio-corona led to a partial masking of the TiO_2_ particles surface and reactivity. Pristine and treated TiO_2_ nanoparticles showed comparable acute toxicity and genotoxicity toward HCT116 cells, whereas a small decrease of the induction of oxidative stress after treatment was observed. *Conclusions:* Overall the results underline the importance of SHDS as a tool to improve the predictive power of in vitro tests towards intestinal nanomaterial toxicity.

## 1. Introduction

Titanium dioxide is a white metal oxide commonly used as whitening and brightening agent in many consumer products such as cosmetic and food goods. As food additive TiO_2_ is referred as E171 and INS171 respectively in EU and US. With these commercial names TiO_2_ is present in many foods including sauce, ice cream and pastries, as well as in the coating of sweets and chewing gum [1,2,3]. In the USA, TiO_2_ content in foods should not exceed 1 wt %, while in the EU it is used “at quantum satis” levels [4,5].

The dietary intake of TiO_2_ has been estimated between 0.2–0.7 mg TiO_2_/kg body weight/day in the USA, 1 mg in the UK [2] and 0.5–1.0 mg in Germany [6]. Children under 10, due to their lower body mass and their higher consumption of candies and sweets compared to the adults are estimated to ingest 1–2 mg TiO_2_ per day in USA and 3 mg TiO_2_ per day in UK [2].

Food grade TiO_2_ consists of particles of 10–350 nm in size [7], containing a fraction of particles in the nano size range up to 36% in number [2,3] with large batch to batch variability [8].

The presence of nanoparticles (NPs) in food-grade TiO_2_ led several laboratories to investigate the possible occurrence of acute or chronic adverse effects following TiO_2_ ingestion.

However, no consensus has been currently reached on the impact of ingested TiO_2_ on human health. Because of these uncertainties, the French Government has prudentially decided to transiently ban TiO_2_ in food from 1 January 2020.

TiO_2_ particles have been shown to cross the intestinal barrier, both in vivo and in vitro, although sometimes translocation was mild [9,10,11,12], and did not show any significant systemic effects [13]. Conflicting data exist on their local effect, where inflammation and enhanced initiation or promotion of colorectal carcinogenesis were observed [14,15,16], but not confirmed [17]. Results from in vitro studies designed to address acute toxicity are contrasting [18], and several long-term or repeated exposure suggest possible chronic effects resulting from the accumulation of TiO_2_ particles in intestinal cells [19,20] whereas other studies suggest the lack of toxic effects and an almost complete elimination in feces [21].

One of the possible sources of discrepancies among data obtained in the different models is the different biological identity of the particles, that depends upon the environment experienced by the particles entering in contact with gut epithelia.

The biotransformation of nanomaterials across the oral–gastro–intestinal (OGI) tract is still poorly understood and only a limited number of studies described this process at a molecular level. [22]. Provided the complexity and variability of the digestive system, dramatic pH and ionic strength changes and the presence of molecules able to interact with the nanoparticle surface are expected to change the biological identity of TiO_2_ and, in turn, affect their toxic potential. Depending upon the kind of nanomaterial, dissolution, variation of the size distribution following agglomeration/de-agglomeration and surface modification (including bio-corona formation) are likely to occur [22].

Recently, Sohal and co-workers described the kinetic of dissolution of different nanomaterials (NMs), including TiO_2_, by using an in vitro system simulating the GI tract [23]. TiO_2_ was found to be highly persistent, reaching the gut with minimal dissolution. Agglomeration/aggregation was observed for several NMs of different chemical nature including TiO_2_ in in vitro systems [23,24,25,26]. However, no aggregation of TiO_2_ was observed in rats following administration by intragastric gavage [27]. Modification of the surface charge likely due to the formation of a protein corona has also been observed [26,28].

The effect of biotransformation in the OGI tract was investigated in vitro by using a Caco-2 cell model [25]. An increase of cytotoxicity was found in digested TiO_2_ as compared to the pristine one, though at very high doses (200 µg/mL).

Food matrix has also an impact on the intestinal toxicity of TiO_2_. Cao and co-workers reported a different cytotoxicity and cell proteome effect of food-grade TiO_2_ depending upon the complexity of the food matrix by using an in vitro system [28]. Moreover, an in vivo study showed immune disturbance and both initiation and promotion of colorectal cancer in rats exposed to E171 [16], although these effects were not further confirmed [17]. The main difference between these two studies is E171 administration via the drinking water in Bettini et al. [17], while it is included in a food matrix in Blevins et al. Again, this highlights the role of the food matrix as a modulating factor of TiO_2_ toxicity.

Overall, the data currently available suggest dramatic changes of properties of ingested TiO_2_. However, the poor understanding of the molecular processes underlying the biotransformation occurring in the OGI tract crossing, hampers the development of appropriate models to simulate the process and the development of in silico methodologies.

In the present study we aimed to get insights into the molecular processes governing the particles bio-transformation in term of agglomeration and surface modifications of TiO_2_ during the digestion process. Fluids at increasing degree of complexity were applied to build an in vitro simulated human digestive system (SHDS). A sample of food-grade TiO_2_ (TiO_2_-FG), one aggregated nanometric TiO_2_ sample (TiO_2_-NM1) and one monodisperse nanometric TiO_2_ sample (TiO_2_-NM2) simulating the nanometric fraction of food grade TiO_2_ were tested.

Not treated and digested TiO_2_-NM1 were compared for their toxicity toward HCT116 cells, assessing their cyto-, genotoxicity and potential to induce oxidative stress as endpoints.

## 2. Materials and Methods

### 2.1. Nanomaterials

The food grade titanium dioxide (Kronos 1171)—here referred as TiO_2_-FG—was purchased by Kronos, Dallas, TX, USA. JRCNM01001a (alias NM-101)—referred here as TiO_2_-NM1—was obtained by the Nanomaterials Repository, European Commission—JRC IHCP, Ispra, Italy.

The sample referred as TiO_2_-NM2 is a water-based dispersion of fumed titanium dioxide (AERODISP^®^ W 740 X) and was purchased by Evonik Industries AG, Essen, Germany. AERODISP^®^ W 740 X is a highly filled dispersion of titanium dioxide AEROXIDE^®^ TiO_2_ P 25 produced by the same company.

In Table 1 the main properties of the samples are reported.

Ultrapure water was obtained from a MilliQ Plus system (Millipore, Bedford, MA, USA) and was always used freshly prepared. All chemicals and solvents used were at least of analytical grade. When not otherwise specified, reagents were purchased from Sigma-Aldrich.

#### 2.1.1. Specific Surface Area

The surface area of the particles was measured by means of the Brunauer–Emmett–Teller (BET) method based on N_2_ adsorption at 77 K (Micrometrics ASAP 2020).

#### 2.1.2. AF4/DLS Analysis

A AF2000 Asymmetric Flow Field Flow Fractionation (AF4) system (Postnova Analytics), incorporating a flat separation channel of 280 mm length, an autosampler (PN5300), an UV-Visible detector (SPD-20A, Shimadzu, Kyoto, Japan) and a dynamic light scattering (DLS) detector (Zetasizer, Nano ZS Malvern Instruments, Malvern, UK) was used for the measurement of the size distribution of titanium dioxide nanoparticles. The AF4 channel was equipped with a regenerated cellulose membrane of 10 kDa molecular weight cut-off and a spacer of 350 µm.

Focusing conditions: focusing time of 7 min, injection flow rate of 0.2 mL/min and crossflow rate of 0.75 mL/min. Elution conditions: exponentially decreasing (exponent 0.2) crossflow rate from 0.75 mL/min to 0 mL/min during 30 min. The detector flow rate was kept at 0.5 mL/min during the focusing and elution steps. The sample injection volume was 20 µL. UV-Visible detection was performed at 254 nm.

### 2.2. Treatment of TiO_2_ Samples with the Simulated Human Digestive System (SHDS)

#### 2.2.1. Preparation of Fluids

Simulated digestive fluids were prepared based on the protocol previously reported by to Sohal et al., 2018. The composition of the fluids is reported in the Supplementary Materials (Table S1).

Briefly, two solutions containing the inorganic and organic components in MilliQ water for saliva, gastric and intestinal fluid were prepared separately and stirred for 24 h. Just before the experiment the two solutions were mixed in a ratio of 1:1 and the pH was adjusted to the typical values reported for healthy adults with empty stomach [30] with 1 M NaOH and 1 M HCl. Then, a protein mix specific for each solution was added and stirred for 5 min.

During the digestion experiment the pH was monitored. In the intestinal phase, pH has to be between 6.5 and 7.5 values to simulate this tract so the pH was adjusted to the value of 6.5 with a 1 M solution of NaHCO_3_ [31].

Solution with the same ionic strength or pH of the simulated digestive fluids were also prepared. The ionic strength of each single simulant digestive fluid was calculated with the equation:

$$I=\frac{1}{2}\overset{n}{\underset{}{\sum }}c_{i}z_{i}^{2}$$

*c_i_* and *z_i_* are respectively the concentration and the charge of the ionic species. The calculated values were 0.133 M for saliva, 2.854 M for the gastric fluid, 0.233 M for the intestinal fluid. Solutions with the same theoretical ionic strength were prepared by using NaCl.

The solutions having the same pH of the simulated digestive fluids (6.5 saliva; 1.4 gastric fluid; 8.1 intestinal fluid) were prepared by adjusting the pH of water with NaOH or HCl. Before measurements, 1 mg of sample was suspended in 1 mL of each solution and treated with an ultrasonic bath for 2 min.

#### 2.2.2. Treatment with Single Simulated Digestive Fluids

The TiO_2_ samples were suspended in MilliQ water (1 mg/mL) and the suspension sonicated using a probe sonicator (Bandelin sonopulse mini 20) for 5 min at 40% amplitude. 1 mL of the mother suspension was diluted to 10 mL with each simulated digestive fluid preheated at 37 °C, obtaining a final concentration of the suspension equal to 100 μg/mL. The suspensions were stirred at 37 °C for 5 min (saliva) or 2 h (gastric or intestinal fluids).

#### 2.2.3. Simulated Digestion Cascade

The TiO_2_ samples were suspended in MilliQ water (1 mg/mL) and the suspension sonicated using a probe sonicator (Bandelin sonopulse mini 20) for 5 min at 40% amplitude. 1 mL of the suspension was diluted to 10 mL with simulated saliva preheated at 37 °C. The suspension of TiO_2_ particles was incubated for 15 min under stirring at 37 °C. 1 mL of the suspension was diluted to 3 mL with simulated gastric fluid and incubated for 4 h under stirring at 37 °C. 1 mL of the suspension was diluted to 2.5 mL with the intestinal fluid and the pH adjusted to the value of 6.5 with a solution of NaHCO_3_ 1 M (obtaining a final concentration of powder equal to 13.2 μg/mL) then and stirred at 37 °C for 4 h

To prepare the samples for cells experiments, the procedure was modified to increase the amount of sample recovered. In this case, a final concentration of 1 mg/mL was obtained.

#### 2.2.4. Reversibility of the Biotransformation

2 mL of the suspension of the TiO_2_ samples in the digestive fluids were centrifuged at 11,000 RPM for 10 min. The supernatant was removed, and the pellet was washed for 3 times with 1 mL of MilliQ water and then suspended again in 1 mL in a bath sonicator for two minutes.

### 2.3. Dynamic Light Scattering Analysis

The mean hydrodynamic diameter and polydispersity index (PDI) measured using a dynamic light scattering (DLS) analyzer (Zetasizer Nano-ZS, Malvern Instruments, Worcestershire, UK) equipped with a HeNe laser operating at 633 nm. Instrument setting: replicates 5, delay time 0, equilibrium time 120 s, T = 25 °C, Dispersant refractive index, and viscosity in solution: 1.330/0.8872 mPa (water); Material refractive index and absorption: 2.490/0.100 (TiO_2_). The measurements were performed after checking that the automatic attenuator was between 7 and 9 and the intercept autocorrelation function < 0.9, according to what recommended by the EUNCL method EUNCL-PCC-001. Three independent replicates were performed for each condition.

The results were expressed as hydrodynamic diameters distribution in intensity (average of mean values of 5 measurements obtained in three independent experiments, i.e., 15 total), mean hydrodynamic diameter (Z-average) and polydispersity index (PDI) ± standard deviation.

### 2.4. Electrophoretic Light Scattering Analysis

ζ-potential was measured by electrophoretic light scattering (ELS) (Zetasizer Nano-ZS, Malvern Instruments, Worcestershire, UK). The samples were suspended in water (0.5 mg/mL) and the ζ potential measured at different pH values (2–9) by adding HCl (0.1 M) or NaOH (0.1 M). The results are reported as mean values of three independent experiments.

### 2.5. Geometrical Diameter Distribution Measured by Flow Particle Imaging Analyzer

Flow Particle Imaging measurements were performed by using a Sysmex FPIA3000 analyzer. Before the measurements, the samples were always washed to avoid protein adsorption on the FPIA tubing system. High power field (2× secondary lens) was applied which allows to measure particles from 1 to 40 μm.

### 2.6. Surface Reactivity

The surface reactivity was monitored by electron spin resonance (EPR) spectroscopy (Miniscope 100 EPR spectrometer, Magnettech, Berlin, Germany), by using 5,5-dimethyl-1-pyrroline-N-oxide (DMPO, Enzo Life Sciences Inc., Farmingdale, NY, USA) as spin trap following a protocol previously developed [32]. To 200 µL of a suspension of TiO_2_ in water, duodenal fluid or cell media (1 mg/mL), 250 µL of DMPO 170 mM and 250 µL of sodium formate 2 M were added, and the suspension constantly stirred in a glass vial under indoor illumination. The EPR spectra were recorded on a sample suspension (50 µL).

Instrument settings: microwave power 7 mW, modulation amplitude 1 G, scan time 80 s, two scans. The negative controls were, in all experiments, the solutions without TiO_2_.

### 2.7. Cellular Experiments

TiO_2_-NM1 was selected to test the effect the simulated digestion on the toxicity of nano-TiO_2_ on human colorectal carcinoma HCT116 cells.

#### 2.7.1. Preparation the Suspensions in Cell Media

Digested TiO_2_ was directly diluted in the McCoy’s 5a medium. Pristine TiO_2_ was dispersed by using the Nanogenotox dispersion protocol as previously described [33]. Briefly, the TiO_2_ samples were pre-wetted in 0.5% EtOH, the diluted in 0.05% bovine serum albumin to obtain a 2.46 mg/mL TiO_2_ suspension. This suspension was dispersed using high energy probe sonication for 16 min with an energy input of 3136 MJ/m^3^, in a water/ice bath.

#### 2.7.2. Cell Culture

HCT116 cells, derived from a human colorectal carcinoma, were purchased from the European Cell Culture Collection (ECACC, Salisbury, UK) and grown in McCoy’s 5a medium to which was added 50 U/mL of penicillin, 50 µg/mL streptomycin and 10% (*v/v*) fetal bovine serum (FBS), at 37 °C, 5% CO_2_ in a humidified atmosphere, a passed twice a week. They were checked for mycoplasma contamination once a week. For toxicity experiments, they were seeded at 20,000 cells per well (WST-1 and DHR123 assays) or 5000 cells per well (53BP1 assay) in 96-well plates. They were exposed to 0–100 µg/mL TiO_2_ NPs (WST-1) or 10, 20, 50 µg/mL TiO_2_ NPs (DHR123 and 53BP1 assays). Positive controls were 100 µg/mL polystyrene-amine NPs (WST-1), 250 µM H_2_O_2_ (DHR123) or 100 µM etoposide (53BP1).

#### 2.7.3. WST-1 Assay

After exposure, exposure medium was discarded and replaced by 100 µL of WST-1 solution (Roche, Basel, Switzerland) diluted to the tenth in FBS-free cell culture medium. After 90 min of exposure at 37 °C, to avoid any optical interference of the NPs with the assay the plates were centrifuged and 50 µL of supernatant was sampled and transferred to a clean plate. Absorbance was measured at 540 nm and corrected by subtraction of background absorbance at 690 nm.

#### 2.7.4. DHR123 Assay

Cells were incubated for 45 min at 37 °C with 1 µM of DHR123 (Thermo Fisher Scientific, Waltham, MA, USA) prepared in phosphate saline buffer (PBS). They were then rinsed and exposed to TiO_2_ particles. The onset of rhodamine fluorescence at λ_exc_/λ_em_ 505/540 nm, reflecting cleavage of DHR123 by intracellular reactive oxygen species (ROS) was then monitored at 30 min, 1, 3, 5, 7, 24 and 48 h post-exposure.

#### 2.7.5. 53BP1 Immunostaining and Foci Count

After incubation for 24 h with TiO_2_ NPs, cells were fixed with 4% formaldehyde, pH 7.4, permeabilized with 0.2% triton X-100 and washed three times with PBS containing 4% non-fat dry milk (washing buffer). They were then exposed to anti-53BP1 antibody (Abnova, PAB12506, dilution 1/500, Taipei, Taiwan) for 1 h at room temperature under mild agitation, rinsed three times for 5 min with washing buffer and incubated for 1 h at room temperature with goat anti-rabbit IgG-Atto 488 (Sigma-Aldrich, 18,772, dilution 1/2000, St. Louis, MO, USA). They were further rinsed three times in washing buffer and counterstained with 5 µg/mL Hoechst 33,342 for 15 min at room temperature. Cells were finally washed three times with PBS, and plates were stored at 4 °C in the dark until analysis using a CellInsight CX5 High Content Screening platform (Thermo Fisher Scientific).

#### 2.7.6. Statistical Analysis

Experiments on cells were reproduced three times independently (n = 3), with 5 technical replicates per independent experiment. Results from these 5 technical replicates were averaged, and the data reported in the figure are the average ± standard deviation of the averages calculated from the three independent experiments. As assumption for normality and homoscedasticity of data could not be verified due to too low number of independent replicates, non-parametric assays were used for statistical significance assessment, i.e., Kruskall–Wallis one-way ANOVA followed by pairwise comparison using Mann-Whitney test. These tests were performed using Statistica version 7.1 (Statsoft).

## 3. Results

### 3.1. Size Distribution and ζ-Potential of the TiO_2_ Samples in Water

**Figure 1:**
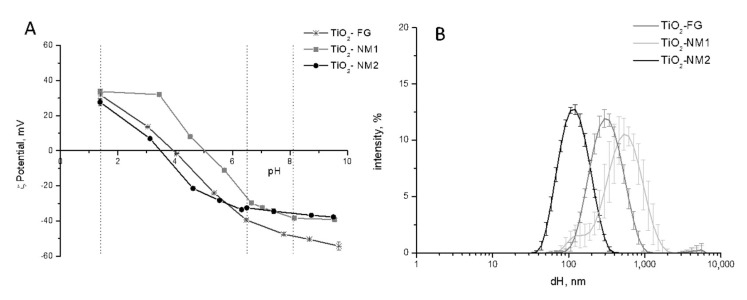
ζ-Potential and size distribution of pristine TiO_2_ samples in water. (**A**) ζ-Potential vs. pH, expressed as mean value of three independent measurements ± SD. (**B**) Hydrodynamic diameters (d_H_) distribution (% intensity) expressed as mean value of 5 measurements in three independent experiments ±SD.

The hydrodynamic diameter distribution and the ζ-potential in the 1–10 pH range of the three TiO_2_ samples suspended in water were firstly measured (Figure 1).

As expected, all TiO_2_ samples exhibited positive ζ-potential at acidic pH and negative ζ-potential at basic pH (Figure 1A). Different point of zero charge were observed, possibly due to the presence of impurities at the surface. For example, for TiO_2_-NM2 several metallic impurities have been detected [29]. The values of ζ-potentials at pH typical of the OGI compartments (vertical lines) were slightly different, in particular at pH 8.5, with a more negative ζ-potential for TiO_2_-FG.

In Figure 1B the hydrodynamic diameters distribution of the three samples is reported. TiO_2_-NM2 appeared monodisperse, with a mean diameter of 107.6 ± 3.166 nm (PdI 0.150 ± 0.005) close to that of the primary particles (Table 1) and with a large fraction in the nanometric range. The size distribution and mean diameter of TiO_2_-NM2 was confirmed by AF4/DLS analysis (Supplemetary Material, Figure S2).

In agreement with what was previously reported [29,34] TiO_2_-NM1 formed in water suspensions aggregates/agglomerates in a range of size from 100 to 1000 nm, while the primary particles size is of few nanometers (Table 1).

As expected, the size distribution of TiO_2_-FG was higher by respect of the other two samples, and the suspension was poorly stable as inferred by the large error bars. Clear sedimentation was visible during time (Supplementary Material, Figure S3).

### 3.2. Size Distribution Changes during the Simulated Digestion Cascade

In the simulated human digestive system (SHDS) the samples are successively in contact with three solutions that simulate the oral, gastric and duodenal environment. The composition of the simulated digestive fluids including inorganic, organic and active components (proteins) are described in the supplementary material (SI Table S1). The digestion cascade was set-up as described in the methods section.

**Figure 2:**
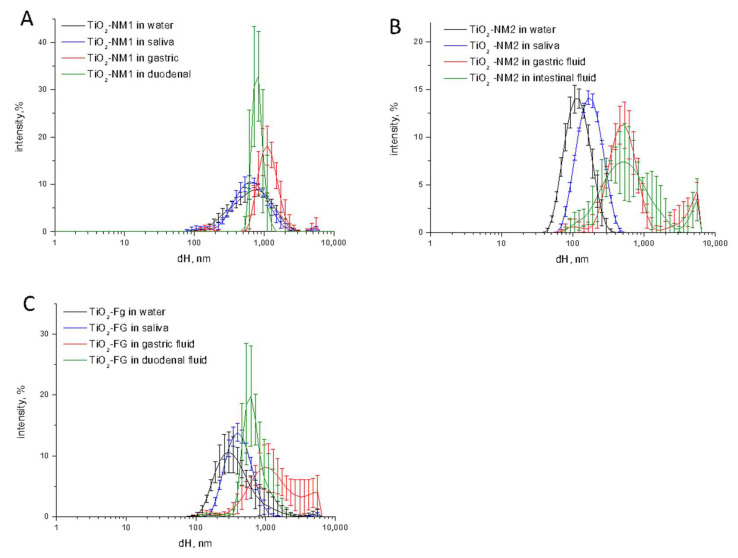
Size distribution monitored by DLS of the TiO_2_ samples during the digestion cascade. (**A**) TiO_2_-NM1; (**B**) TiO_2_-NM2; (**C**) TiO_2_-FG. Hydrodynamic diameters (d_H_) distribution (% intensity) is expressed as mean value of 5 measurements in three independent experiments ±SD.

In Figure 2 the size distribution of the three TiO_2_ samples in saliva, gastric fluid and intestinal fluid during the digestion cascade monitored by DLS is compared with the size distribution of the samples in water.

Small or no changes in size distribution was observed in saliva for both food-grade and nanometric TiO_2_ samples. Conversely, a dramatic increase of the hydrodynamic size was observed in simulated gastric fluid. In all cases the agglomeration/aggregation was observed also in the simulated duodenal fluid. This is inferred not only by the shift of the curve toward higher d_H_ values, but also by the increase of the variability among the five measurements (error bars), suggestive of a poor stability of the suspensions. Consistently, sedimentation was visually observed (Figure S2). A residual nanometric fraction was observed for the TiO_2_-NM2 sample only. Note however that DLS underestimate the smaller fractions due to the lower intensity of the scattered light. The presence of nanometric particles cannot therefore be excluded.

**Figure 3:**
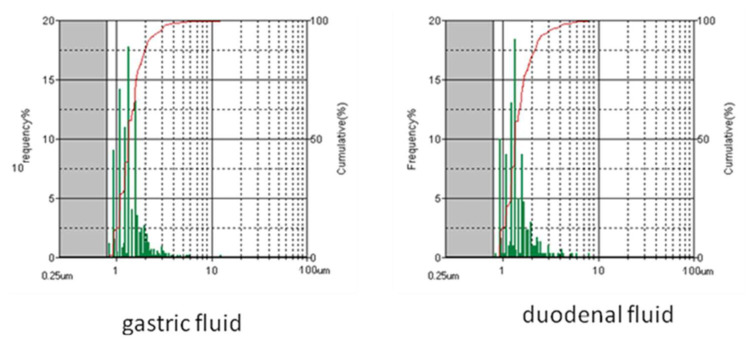
Size distribution (green) and cumulative size distribution (red) for TiO_2_-NM1 in vitro digestion cascade measured by the flow particle imaging analyzer.

The presence of aggregates having diameter higher than that detected by DLS (>2 μm) was monitored by using the flow particle imaging analyzer on TiO_2_-NM1 in the gastric and duodenal fluids (Figure 3). This technique detects the presence of agglomerates/particles from 1 to 300 μm.

Similar size distribution was observed in the gastric and duodenal fluid. The geometrical diameters were distributed in the 1–4 and 1–5 μm ranges respectively, with the 80% of the particles having a diameter lower than 2 μm in both fluids. Note that the analysis is performed on samples washed with water and in a fluid containing a surfactant. Therefore, it can detect the presence of aggregates and not agglomerates, since size of the latter are expected to be media dependent (see below).

### 3.3. Irreversibility of Size Distribution Changes

**Figure 4:**
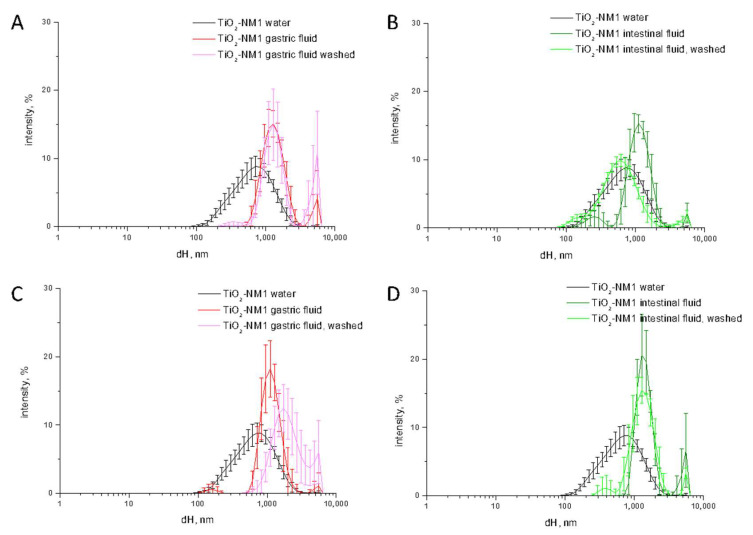
Size distribution monitored by dynamic light scattering (DLS) of TiO_2_-NM1 incubated in the single simulated fluids (**A**,**B**) or after the digestion cascade (**C**,**D**). The measurements were performed on the treated sample and on the sample treated and washed with water. The hydrodynamic diameters (d_H_) distribution (% intensity) is expressed as mean value of 5 measurements in three independent experiments ±SD.

Agglomeration is, by definition (EU Recommendation 2011 696/EU), a reversible process, that depends upon the environment. Conversely, aggregation, i.e., the formation of strong bonds between particles, is an irreversible process. Aggregated particles are expected to remain therefore unaltered moving from one OGI tract compartment to the other. To investigate the nature of the agglomeration/aggregation process observed in the SHDS (Figure 2) the size distribution was measured on TiO_2_-NM1 treated in the single gastric and duodenal fluids or in cascade. Part of the sample was washed and re-suspended in water prior DLS measurements. The data are compared in Figure 4.

The size distribution of TiO_2_-NM1 treated with the gastric fluid washed with water was overlapped with those observed for the sample not washed (Figure 4A) suggesting that the agglomeration in the gastric fluid was irreversible. Conversely, in the duodenal fluid the size distribution after washing resemble those of the pristine sample, indicating that the agglomeration process was reversible (Figure 4B).

On the other hand, while a similar trend was observed in the gastric fluid in the cascade (Figure 4C), no modification of the size distribution was observed after washing in duodenal fluid (Figure 4D). This suggests that in the gastric fluid irreversible aggregation occurs, and that the particle size remains unaltered in the duodenal compartment.

### 3.4. Effect of the pH, Ionic Strength, and Phosphate Ions on Aggregation/Agglomeration

Agglomeration may be a consequence of a reduction of the electrostatic repulsion among particles. This may be in turn due to a decrease of the surface charge following pH and ionic strength modification, or adsorption of the components of the fluids at the surface of the particles.

**Figure 5:**
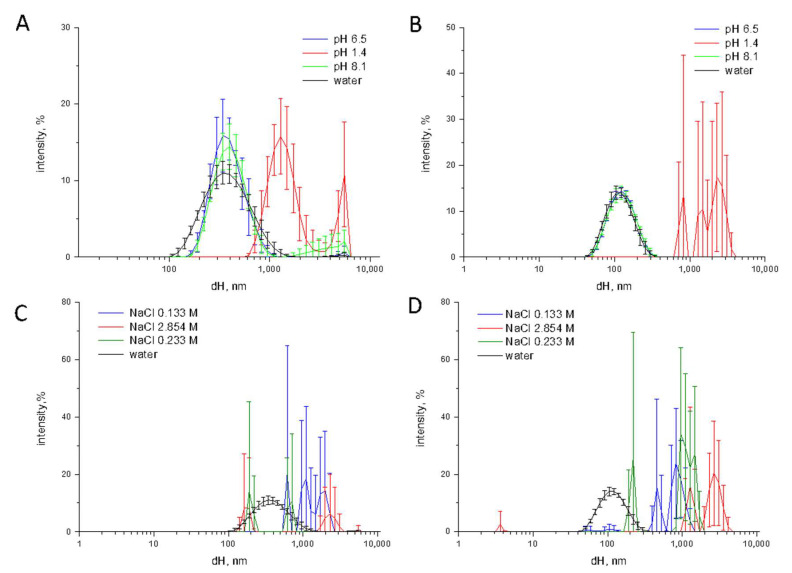
Effect of pH and ionic strength on size distribution of TiO_2_ samples. Size distribution monitored by DLS of (**A**) TiO_2_-FG; and (**B**) TiO_2_-NM2 in water at pH 6.5, 1.4 and 8.1; size distribution of (**C**) TiO_2_-FG; and (**D**) TiO_2_-NM2 in solutions of NaCl with different ionic strength.

In order to investigate the influence of the pH on particle agglomeration, TiO_2_ samples were suspended in MilliQ water with a pH correspondent to the values of the OGI compartments, i.e., pH 6.5 in saliva, pH 1.4 and pH 8.1 in gastric and duodenal fluids, respectively (Figure 5).

No significant variation in size distribution was observed when the samples were suspended in a solution with neutral or basic pH (Figure 5A,B). Conversely, at the strongly acidic pH of the gastric milieu the mean d_H_ and the PDI largely increases for TiO_2_-NM2 and TiO_2_-FG. This was not expected since at acidic pH the particles exhibit a positive surface charge and a high ζ-potential (Figure 1), suggesting ionic strength as the driving force.

This was confirmed by preparing solution of NaCl at ionic strength equal to the theoretical ionic strength of saliva (0.133 M), gastric fluid (2.854 M) and duodenal fluid (0.233 M). A dramatic increase of the hydrodynamic diameter was observed in all suspensions, that became very unstable as inferred by the large error bars. This was observed also on the NaCl solution having the ionic strength of saliva, while no change of size was found in the simulated saliva. One possible reason for this discrepancy, is the overestimation of the calculated ionic strength. In fact, calculation (Method section) considers all the electrolytes as completely dissociated. Therefore, the real ionic strength of simulated fluids may be lower than the correspondent NaCl solution.

**Figure 6:**
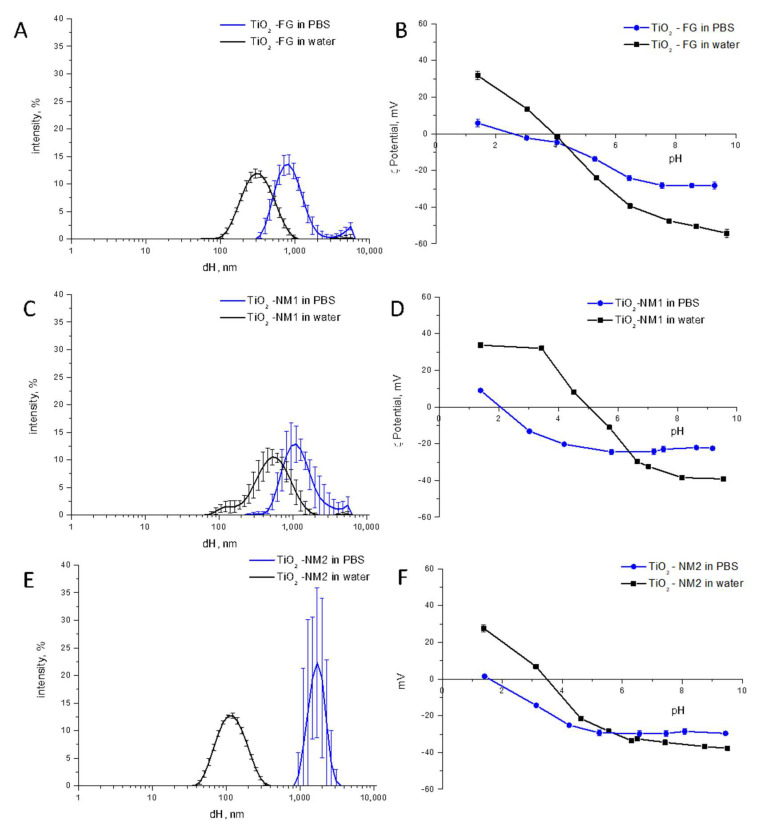
Effect of phosphate ions on the size distribution and ζ-potential of TiO_2_ samples. Left panels: ζ-potentials vs. pH, expressed as mean value of three independent measurements ± SD. Right panels: Size distribution monitored by DLS. (**A**,**B**) TiO_2_-FG; (**C**,**D**) TiO_2_-NM1; (**E**,**F**) TiO_2_-NM2.

We previously reported that phosphate ions strongly adsorb on the surface of TiO_2_ by decreasing the ζ-potentials [35]. In order to assess the role of phosphate ions on TiO_2_ aggregation, the samples were suspended in a 10 mM saline phosphate buffer (PBS) and the hydrodynamic diameter was measured (Figure 6).

Strong particle agglomeration/aggregation in PBS was observed, an effect that might be due to a modification of the ζ-potentials following adsorption of phosphate ions. An effect of the ionic strength may be excluded since the value for PBS (0.15 M as declared by the company), is similar to those of the saliva. Comparing the two nanometric samples, the effect is more evident on the monodispersed TiO_2_-NM2. This was expected since TiO_2_-NM1 was already largely aggregated in water.

The ζ-potentials of TiO_2_-NM2, TiO_2_-FG and TiO_2_-NM1 in a range of pH from 2 to 9 in PBS was measured and compared with those in water (Figure 6, right panels). As already reported for another TiO_2_ sample [36], a shift toward more acidic pH of the points of zero potential was observed for the three samples, due to the adsorption of phosphate ions at the surface. At neutral pH a decrease of the ζ-potentials was observed, in agreement with the agglomeration measured by DLS.

### 3.5. Bio-Corona Formation

When NPs interact with biofluids the formation of a bio-corona is likely. The bio-corona components, mainly proteins, may interact with the surface reversible or irreversibly.

Here, proteins appeared to be involved in the bio-corona formation. In fact, by performing the digestion cascade by using fluids without proteins (SI, Figure S1) the suspensions were much less stable than in the case of the digestion cascade with proteins (Figure 2) in gastric and duodenal fluid, since particles lack of the stabilizing effect of proteins.

**Figure 7:**
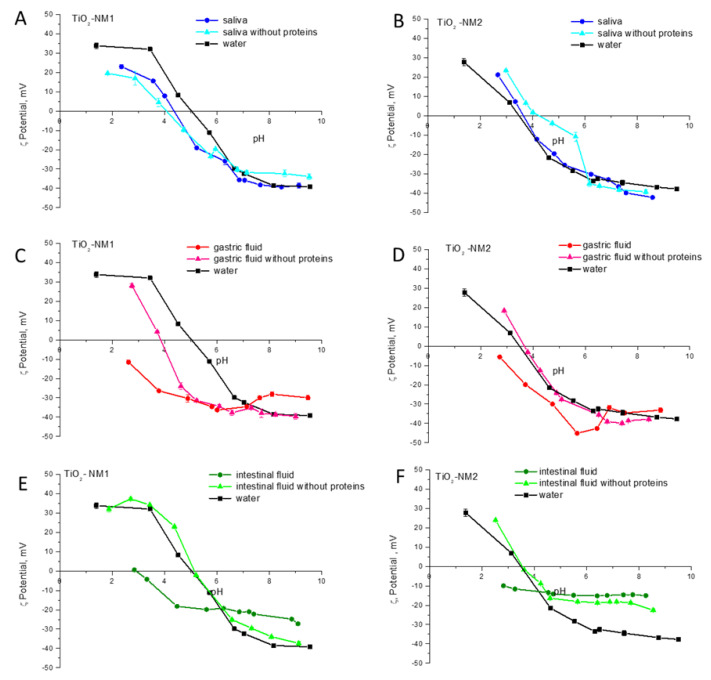
Effect of the hard-biocorona on the ζ-potential of the nanometric TiO_2_ samples. ζ-potential vs. pH measured in water of the pristine samples and of the samples treated in saliva, gastric and duodenal with and without proteins and washed to remove molecules weakly adsorbed. (**A**,**C**,**E**): TiO_2_-NM1; (**B**,**D**,**F**): TiO_2_-NM1.

In order to verify the reversibility of protein-surface interaction, the samples were incubated in the saliva, gastric and duodenal fluids with and without proteins and then washed for three times to remove the soft corona. The ζ-potential of treated samples was compared with those of the pristine one (Figure 7).

Small differences were observed for the samples suspended in water and in simulant saliva with and without proteins. Conversely, the ζ-potential curves of the samples treated in gastric and in duodenal fluids with proteins were different from those suspended in water, while in the absence of proteins the curves were closer, albeit not overlapped, to those recorded on the samples incubated in water. Since the samples were washed prior the measurement to remove molecules weakly adsorbed at the surface, these data suggest the formation of a hard-biocorona in gastric and duodenal fluid, containing proteins.

### 3.6. Characterization of TiO_2_-NM1 in Cell Media

**Figure 8:**
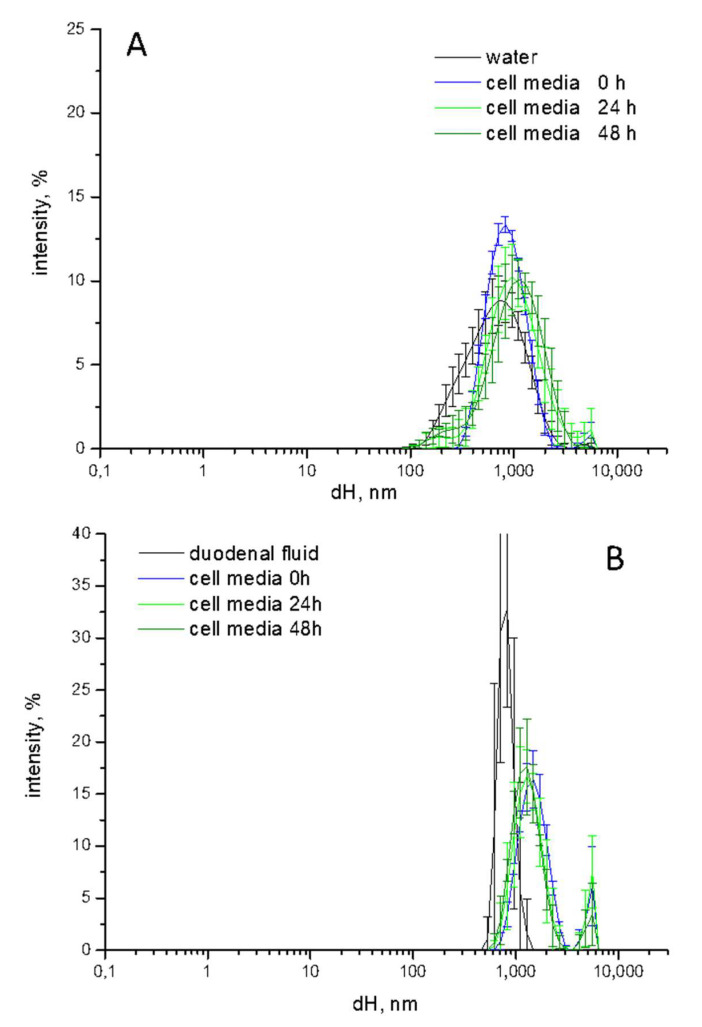
d_H_ distribution of (**A**) pristine and (**B**) pre-treated TiO_2_-NM1 during the incubation in McCoy’s 5a medium + fetal bovine serum (10%). Hydrodynamic diameters (d_H_) distribution (% intensity) is expressed as mean value of 5 measurements in three independent experiments ±SD.

Once diluted in the cell media TiO_2_ particles are expected to undergo further modifications following the interaction with the cell components and serum proteins added to the medium. To monitor the process, the agglomeration state of TiO_2_-NM1 pristine or pre-treated with the SHDS was evaluated by DLS. In Figure 8 the d_H_ distribution of (A) pristine and (B) pre-treated samples during the incubation in the cell media are reported (Figure 8).

The pristine sample (Figure 8A) appeared slightly more agglomerated/aggregated in cell media than in water. Small changes in size distribution was observed during 48 h of incubation, indicating that the suspension was stable during time. A moderate increase in size was also observed for the pre-treated sample after dilution in cell media. Additionally, in this case the suspension was stable during time. The comparison of the size distribution of pristine vs. pre-treated samples reveals the disappearance of the nanometric fraction after digestion.

**Figure 9:**
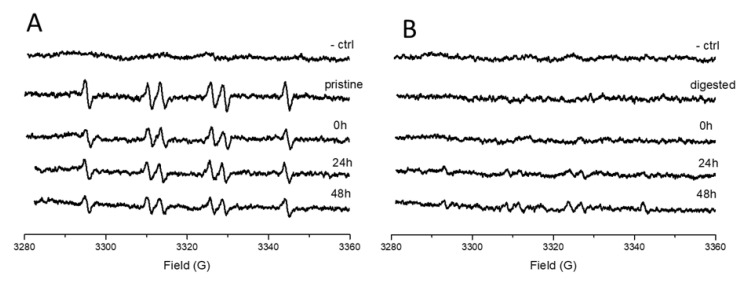
Effect of digestion and cell media incubation on the surface reactivity of TiO_2_-NM1. EPR signals recorded after 30 min of incubation under indoor illumination of (**A**) pristine TiO_2_-NM1 and (**B**) digested TiO_2_-NM1 during the incubation in McCoy’s 5a medium + fetal bovine serum (10%) monitored by electron spin resonance (EPR) spectroscopy.

ELS results showed that the digestion process induces the formation of a hard bio-corona. However, the data do not reveal whether the TiO_2_ surface is partially or completely coated. The intrinsic photocatalytic activity of TiO_2_ was exploited as probe to investigate this aspect. The surface photo-reactivity of pristine or digested TiO_2_-NM1 was monitored before and after incubation in the cell media (Figure 9) by EPR spectroscopy associated to the spin trapping technique [32].

As expected, the untreated sample (Figure 9A) generated a six-line EPR signal correspondent to the adduct DMPO–COO^•−^ that is generated by the reaction of sodium formate with the surface of TiO_2_ activated by UV light [32]. The reactivity slightly decreased following incubation in the cell media, suggesting the formation of a bio-corona that left part of the surface still exposed to the solvent. A complete suppression of the surface reactivity was observed for the sample after the treatment with the simulated digestive system (Figure 9B) thus confirming the formation of a hard protein corona. After 48 h of incubation in cell media, the surface reactivity was partially restored, indicating the ability of the media constituents to modify the bio-corona previously formed in SHDS.

### 3.7. Cytotoxicity, Genotoxicity and Oxidative Stress on HCT116 Cells Exposed to Pristine or Treated TiO_2_-NM1

**Figure 10:**
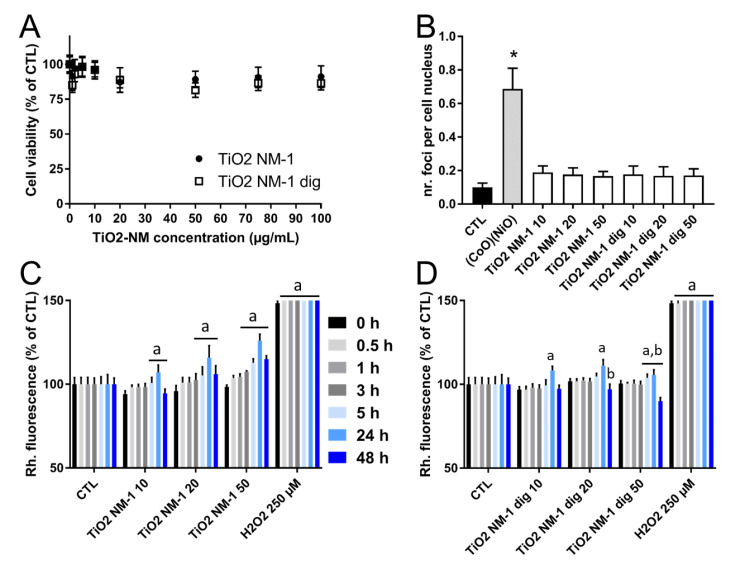
Toxicity of untreated and digested TiO_2_-NM1. Cytotoxicity of TiO_2_-NM1 assessed using the WST-1 assay (**A**); genotoxicity assessed using 53BP1 immuno-labelling and foci count (**B**); oxidative stress assessed via DHR123 assay (**C**). Cells were exposed for 24 h (**A**,**B**) or 0.5 to 48 h (**C**,**D**) to pristine or digested TiO_2_-NM1. Positive control: 100 µg/mL PS-NH_2_, resulting in 80% decrease of metabolic activity in the WST-1 assay (not shown), 250 µM H_2_O_2_ in the DHR123 assay, 100 µM etoposide in the 53BP1 assay. Statistical significance: a: *p* < 0.05, 0.5 or 1 or 3 or 5 or 24 or 48 h vs. 0 h; b: *p* < 0.05 TiO_2_-NM1 pristine vs. TiO_2_-NM1 after SHDS, * *p* < 0.01 TiO_2_-NM1 pristine vs. TiO_2_-NM1 after SHDS.

The toxicity of TiO_2_-NM1 after simulated digestion was compared to that of untreated TiO_2_-NM1 in HCT116 cells (Figure 10).

Although cell viability (Figure 10A) slightly decreased after exposure of cells to the untreated sample, this decrease was statistically not significant. No significant impact of simulated digestion on TiO_2_-NM1 cytotoxicity was observed. Additionally, the genotoxic impact of the two sample was essentially similar, with no significant increase of 53BP1 foci counts (Figure 10B). Regarding ROS intracellular level, a time- and concentration-dependent increase in ROS content was observed in cells exposed to untreated TiO_2_-NM1 (Figure 10C). The same trend was observed in cells exposed to TiO_2_-NM1 exposed to SHDS (Figure 10D). However, in the latter case, a decrease of ROS generation at high doses was observed in the sample recovered after the digestive cascade.

## 4. Discussion

Evidence for possible long-term effects of TiO_2_ on human health are rapidly growing. Unfortunately, most of the data obtained in both in vivo and in vitro models ignore the transformation of NM during the OGI tract transport [37] and the effect of such changes on their bioreactivity.

To the best of our knowledge, there are only two studies that integrate in vitro models with systems that simulate the digestion process. In the first, by Song and co-workers, the cytotoxicity toward Caco-2 cells, either non differentiated or differentiated, was evaluated [25], while Cao and co-workers used a more sophisticated tri-culture model of intestinal epithelium (Caco-2/Ht29/MTX/Raji B cells) [28]. However, the latter study was focused on the effect of the food matrix, and no comparison with the pristine material was performed [28]. In both studies, characterization of the material was performed only at the end of the digestion process.

The present study assessed the transformation of food-grade TiO_2_ during the transit in the OGI tract by using a simulated human digestive system (SHDS), with the purpose to better understanding of the molecular mechanism that govern the bio-transformation processes. At the same time, the consequences of such biotransformation on the toxic effect toward HCT116 cells epithelial intestinal cells were investigated.

One food-grade TiO_2_ (TiO_2_-FG) and two TiO_2_ powders (TiO_2_-NM1, TiO_2_-NM2) were tested. The two nanometric powders where chosen to simulate the ultrafine fraction of the food-grade TiO_2_. This fraction appears highly aggregated/agglomerated [7]. However, since the techniques currently used for the evaluation of size do not distinguish between aggregated and agglomerated, one aggregated sample (TiO_2_-NM1) and one monodisperse sample (TiO_2_-NM2) were selected for comparison.

The interaction of NPs with biofluids is characterized by the formation of a bio-corona, a layer of molecules, in particular proteins, adsorbed at the surface of the materials [38,39]. Reversible and irreversible adsorption may occur depending upon the affinity of the molecules for the surface. In a collaborative study, we demonstrated that Aeroxide^®^ P25 TiO_2_ NPs can absorb bioactive molecules (such as endotoxins) on its bio-corona originating from the incubation with FBS 10%, leading to markedly potentiated pro-inflammatory effects on murine macrophages [40].

The OGI tract compartments are characterized by strongly different environmental conditions; therefore, the bio-corona is expected to be modified during digestion. The molecules that interacts with the material irreversibly (hard-biocorona) are more likely be carried across the OGI tract reaching the gut, being therefore the most relevant in term of effect on the gut epithelia.

In agreement with previous studies for other materials [26,41,42], proteins appeared to be involved in the bio-corona formation. In fact, by performing the digestion cascade by using fluids without proteins (SM, Figure S1) the suspensions were much less stable than in the case of the digestion cascade with proteins (Figure 2) in gastric and duodenal fluid, since particles lack of the stabilizing effect of proteins.

The data show that TiO_2_ samples undergo dramatic changes of size distribution due to aggregation or agglomeration processes. Nanometric TiO_2_-NM1 forms at the end of the digestion cascade aggregates smaller than 10 µm, with 80% in number of particles of less than 2 µm. These results confirm what reported in previous studies on TiO_2_ and other NMs [10,23,25,28]. The increase of particles size is a consequence of the aggregation/agglomeration occurring in the simulated gastric compartment, where the high ionic strength appears to be the main driving force of this process. The effect of pH and bio-corona cannot be however ruled out. This is important since ionic strength is expected to act similarly in any colloidal solution, regardless of the kind of NM, while pH acts differently, depending upon the Brönsted acidity of the surface.

The increase in particles size appears to be irreversible, suggesting that, in vivo, the nanometric fraction of TiO_2_ could reach the gut epithelia mainly as micrometric aggregates. Once diluted in the cell media, TiO_2_-NM1 remained substantially aggregated with a size distribution different from those of the pristine one. In fact, the finest fraction (approximately 100–400 nm) disappear whereas the mean d_H_ increases from 800 nm for pristine sample to 1100 nm for the sample pre-treated with SHDS. Such larger particle/aggregates are expected to undergo macropinocytosis [43] and the small d_H_ variation observed here is unlikely to modify the mechanism of uptake by cells, although it would possibly modify the extent of uptake.

Another relevant aspect for hazard assessment is the formation of the bio-corona. Our data demonstrate the formation of a bio-corona that contain phosphate ions and, possibly, other low molecular weight species and proteins. This bio-corona is partially composed by species hardly adsorbed at the surface, that therefore are expected to be involved in the interaction process between particles and intestinal cells. The nature of the protein fraction of the bio-corona was not investigated, however pepsin and mucin are likely present. In fact, pepsin was present only in the gastric fluid, while mucin was present in the gastric fluids and in saliva, but at a concentration 60 times lower than in the gastric fluid. Pepsin was found to interact with silver NPs in a simulated gastric fluid [26] and polystyrene NPs [41]. Interaction between NPs and mucin was also previously reported [42]. The mucin hydrogel is in fact a network consisting of either positively or negatively charged segments that can strongly interact with charged particles and immobilize them in the mucus [42].

The formation of the bio-corona was found to totally suppressed the surface reactivity of TiO_2_, suggesting a complete coating of the surface by the digestive fluids components. However, when incubated in the cell media, some reactivity was observed even after 48 h of incubation, suggesting that the bio-corona was partially removed/modified following the interaction with the cell media components. This is not in contrast with the data showing the irreversibility of the bio-corona in digestive fluids: in fact, irreversibility is a function of the environment surrounding the material.

These results agree with the higher reactivity of pristine TiO_2_ compared to digested TiO_2_, that leads to an increased ROS content in cells exposed to pristine TiO_2_, compared to digested TiO_2_, covered with digestive fluid components. Still, TiO_2_ in contact with SHDS milieu showed some reactivity towards cells, as shown by a slight increase of ROS content after 24 h of exposure to these NPs, which is consistent with the presence of residual reactive sites at the surface of digested particles, as shown by the EPR measurements. These data also suggest that such changes of surface reactivity play a major role in the oxidative stress generated by TiO_2_ particles, and could have consequences on the NPs cytotoxicity and genotoxicity [44]. Conversely, the present data show no significant consequence of the bio corona formation on NPs cytotoxicity and genotoxicity, maybe because cells can resolve this elevation of ROS thanks to intrinsic antioxidant response. Song et al. reported impaired cell proliferation in the live/dead assay, i.e., increased toxicity, when cells are exposed to digested TiO_2_ NPs as compared to pristine TiO_2_ NPs [25]. They also observe decreased viability (86% of control) in the WST-8 assay (which is equivalent to the WST-1 assay used here), in cells exposed to 200 µg/mL digested but not pristine TiO_2_ particles [25]. It is noteworthy that the loss of cell viability reported in Song et al.’s study is similar to that found here. Note however that in our study we used a different statistical analysis, which shows a not significant effect of TiO_2_-NM1, both pristine and aged, on cell viability. Moreover, the biological relevance of a loss of cell viability lower than 15–20% should be interpreted with caution, because the variability of in vitro cytotoxicity assays at these values. Moreover, Song et al. [25] used 200 µg/mL as exposure concentration, which is a higher concentration as compared to the highest concentration used in the present study and the composition of gastrointestinal fluids slightly differs from those used in the present study, with three-fold higher urea, pepsin, pancreatin and lipase concentrations. This would lead to different bio-coronas and consequently different outcomes in the toxicity tests, which could explain the discordant results.

Cytotoxicity and increase of ROS content in a tri-culture model of intestinal cells, composed of Caco-2, HT29-MTX and RajiB cells exposed to digested TiO_2_ was reported by Cao et al. [28]. However, albeit the digestion model was similar to those used in the present study, the TiO_2_ samples were dispersed in a food matrix, while the TiO_2_ concentrations were much higher, i.e., 0.75 and 1.5% (*w/w*) that correspond to 7.5 and 15 mg/mL. This certainly explains the much more toxic effect observed in this study by respect to the present results.

Overall, our data suggest that TiO_2_ NP that acquire a bio-corona by interacting with the SHDS fluids are less bioactive and toxic, as inferred by a decrease of ROS generation in exposed cells. However, this does not affect cell viability likely because cells still have enough capacity to counteract this increase of ROS levels. This might not occur at higher exposure concentrations [25,28] that might overwhelm the antioxidant capacities of cells.

## 5. Conclusions

In conclusion, the data presented herein show that nanometric TiO_2_ particles treated with a SHDS are biologically different from a non-treated material in term of particles size and surface properties, with the formation of a hard-biocorona. This transformation reduces their ability to induce ROS generation in our cell model, while has not effect on cell viability and genotoxicity. This suggest that in traditional in vitro tests nanometric TiO_2_ particles could cause higher oxidative stress as compared to what observed in vivo, while the pre-treatment with a SHDS would make the results more realistic.

## Figures and Tables

**Table 1:** Physical and chemical properties of the TiO_2_ samples investigated.

	TiO_2_-FG	TiO_2_-NM1 ^c^	TiO_2_–NM2 ^d^
**Specific surface area (m^2^/g)**	9 ± 0.45 ^a^	316.07 ± 15.80	35–65
**Primary particle size (nm)**	10–350 ^b^	5–6	70 (mean)
**Aggregate size (nm)**	-	10–170	-
**Crystalline phase**	Anatase (>99%)	Anatase (98.1%)	Anatase (85%); Rutile (15%)
**Impurities**	-	Al, Na, P, S	<0.5%

^a^ Brunauer–Emmett–Teller (BET) method, this study, ^b^ Reference [7], ^c^ Reference [29], ^d^ as declared by the Company.

## Supplementary Materials

The following are available online at https://www.mdpi.com/2079-4991/10/11/2132/s1, Table S1: Composition of simulated fluids; Figure S1: Size distribution monitored by DLS of the TiO_2_ samples during the digestion cascade in the absence of proteins; Figure S2: AF4/DLS fractogram of TiO_2_-S; Figure S3: Appearance of the suspensions of the TiO_2_ samples during the digestion cascade.
