# Supplementary material for: Biotransformation of Food-Grade and Nanometric TiO2 in the Oral–Gastro–Intestinal Tract: Driving Forces and Effect on the Toxicity toward Intestinal Epithelial Cells
Source: Nanomaterials (Basel). 2020 Oct 27;10(11):2132. doi: 10.3390/nano10112132 (PMC7692287; doi:10.3390/nano10112132)
Supplement: Supplementary file 1 [file nanomaterials-10-02132-s001.pdf]

Biotransformation of food-grade and nanometric TiO_2 in the oral-gastro-intestinal tract: driving forces and effect on the toxicity toward intestinal epithelial cells

Arianna Marucco[1], Marion Prono [2], David Beal[2], Enrica Alasonati[3], Paola Fisicaro[3], Enrico Bergamaschi[4], Marie Carriere[2*], and Ivana Fenoglio[1*]

[1] Dept. of Chemistry, University of Torino, 10125-Torino, Italy

[2] Univ. Grenoble Alpes, CEA, CNRS, IRIG, SyMMES, Chimie Interface Biologie pour l'Environnement, la Santé et la Toxicologie (CIBEST), F-38000 Grenoble, France;

[3] Département Biomédicale et Chimie Inorganique, Laboratoire national de métrologie et d'essais, 75724 Paris

[4] Department of Public Health and Pediatrics, University of Torino, 10126 Torino, Italy;

Supplementary materials

Table S1. Composition of simulated OGI fluids

	Simulated saliva	Simulated gastric fluid	Simulated intestinal fluid
**pH**	6.5 ± 0.1	1.4 ± 0.1	8.1 ± 0.1
**Inorganic components (g/L)**	KCl 0.90<br>KSCN 0.20<br>NaH_2PO_4 x H_2O 1.02<br>Na_2SO_4 0.57<br>NaCl 0.30	KCl 0.82<br>NH_4Cl 0.31<br>$CaCl_2$ x $2H_2O$ 0.40<br>NaCl 2.75<br>NaH_2PO_4 x H_2O 0.31	$MgCl_2$ x $6H_2O$ 0.05<br>KCl 0.94<br>KH_2PO_4 0.08<br>$NaHCO_3$ 9.17<br>NaCl 12.27<br>$CaCl_2$ x $2H_2O$ 0.42
**Organic components (g/L)**	Urea 0.20	Urea 0.09<br>D-Glucose 0.65<br>Glucuronic acid 0.02<br>D-Glucosamine hydrochloride 0.33	Urea 0.35
**Active components (g/L)**	Mucin 0.05<br>Uric acid 0.016<br>α - amylase 0.145	Mucin 3<br>BSA 1<br>Pepsin 1	Pancretin 3.00<br>Lipase 0.5<br>Bile 6.00<br>BSA 2.8

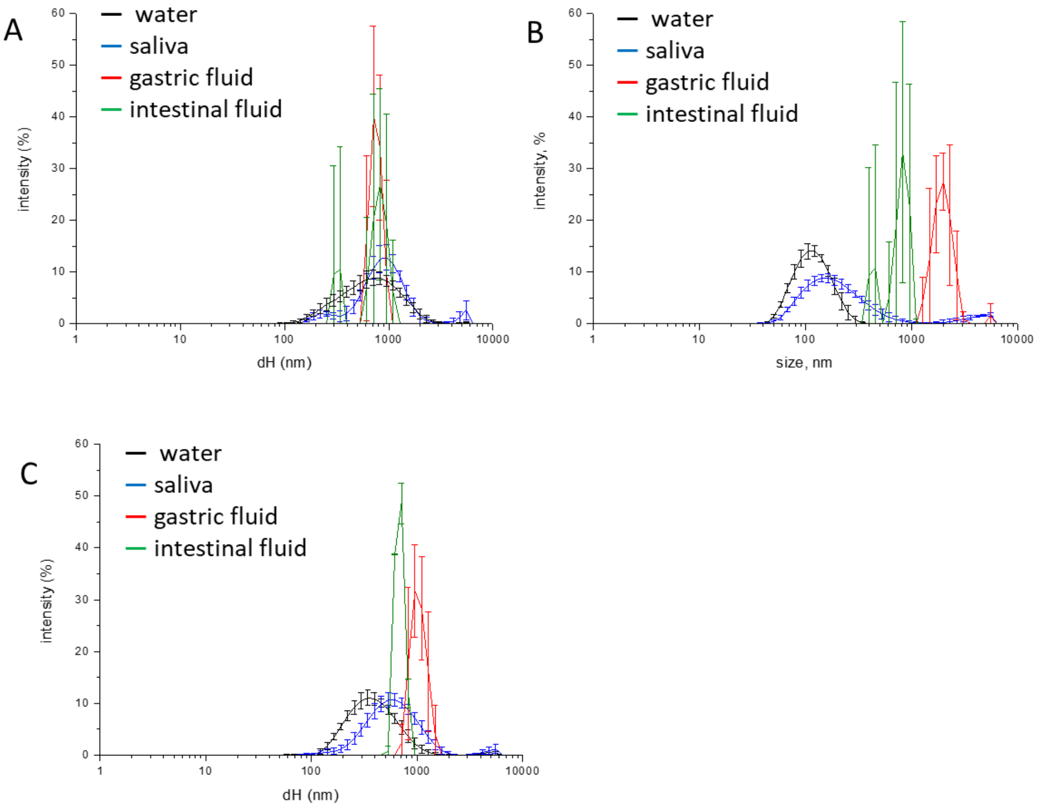

Figure S1. Size distribution monitored by DLS of A) TiO_2-NM1; B) TiO_2-NM2; C) TiO_2-FG during the digestion cascade in the absence of proteins. The hydrodynamic diameters (d_H) distribution (% intensity) is expressed as mean value of 5 measurements in three independent experiments ± SD.

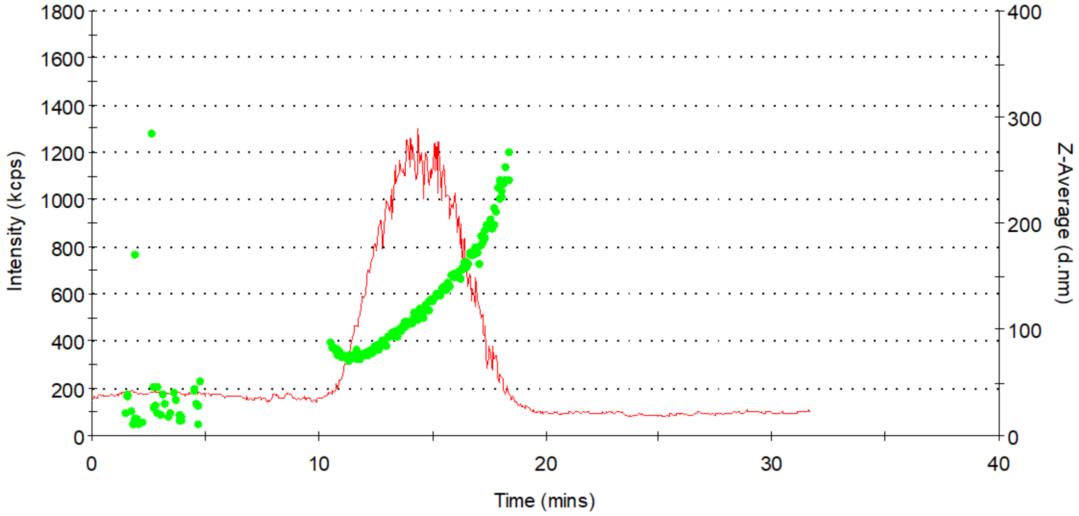

Figure S2. AF4/DLS fractogram of TiO2-S. Concentration of the injected suspension: 50 mg L^{-1}. Mean diameter: 77.3 nm.

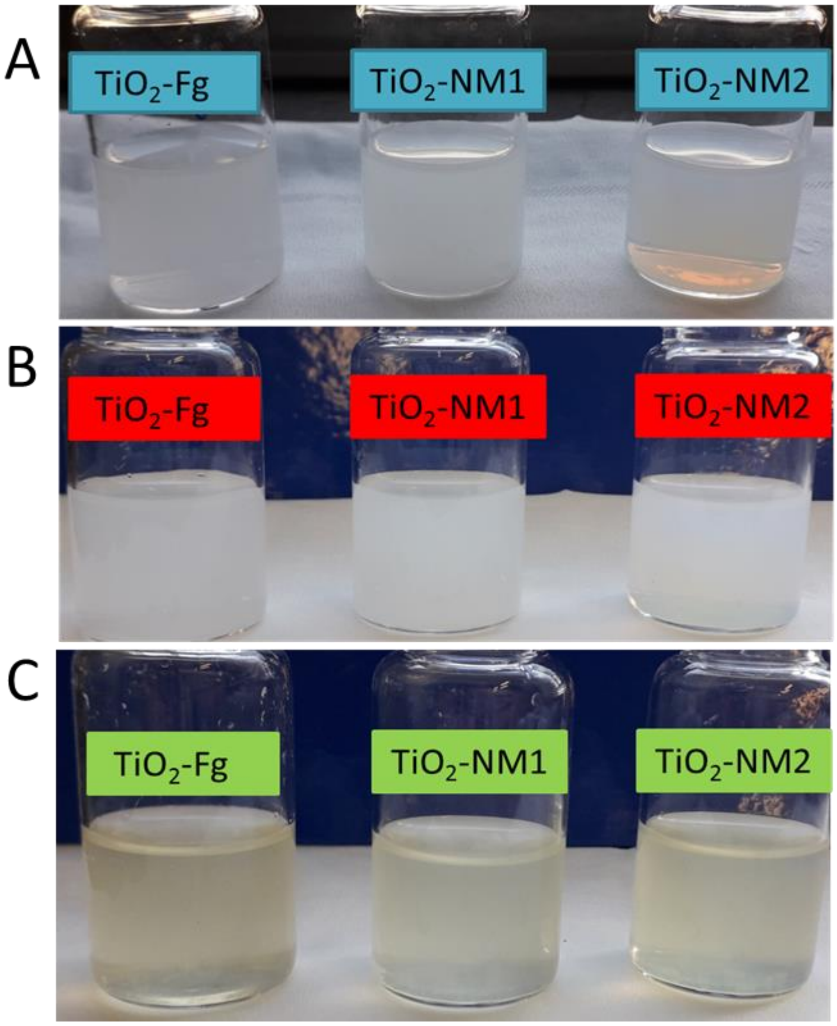

Figure S3. Appearance of the suspensions of the TiO_2 samples during the digestion cascade. A) saliva; B) gastric fluid; C) intestinal fluid.
